# Telomerase activity in T-cells as a functional test for pathogenicity assessment of novel genetic variants in telomere biology disorders

**DOI:** 10.1038/s41598-025-12566-7

**Published:** 2025-08-08

**Authors:** Olivia Carlund, Anna Norberg, Pia Osterman, Ida Andersson, Alva Eriksson, Sofie Degerman, Magnus Hultdin

**Affiliations:** 1 Department of Medical Biosciences, Pathology, Umeå University, Umeå, Sweden; 2 Department of Medical Biosciences, Medical and Clinical Genetics, Umeå University, Umeå, Sweden; 3 Department of Clinical Microbiology, Umeå University, Umeå, Sweden

**Keywords:** Telomere biology disorders, Telomerase activity, Functional analysis, Telomere length, Genetic variants, Disease genetics, Functional genomics

## Abstract

The telomerase enzyme is essential for telomere maintenance. Pathogenic variants in telomere-associated genes have been associated with critical telomere shortening, resulting in telomere biology disorders (TBD) such as bone marrow failure, idiopathic pulmonary fibrosis, and dyskeratosis congenita. The TBDs are clinically heterogeneous and families with TBD often experience an earlier onset and increased symptom severity for each generation. Consensus guidelines have identified certain genetic variants as pathogenic or likely pathogenic, but many are classified as variants of uncertain significance (VUS) in the absence of additional supporting evidence. The pathogenicity of a VUS in genes encoding the telomerase complex could be evaluated by in vitro telomerase activity (TA) measurement. We have developed a functional TA assay in patient-derived T-cells based on the Telomeric Repeat Amplification Protocol (TRAP) combined with qPCR. TA was significantly lower in six TBD patients with a *TERT* or *TERC* variant compared to controls (0.11 versus 0.54, *p* < 0.001). Four patients had a TA of more than three standard deviations below the mean of controls, strongly supporting pathogenicity of the variants. In summary, functional analysis of TA in patient-derived cells could support pathogenic evaluation in clinical diagnostics and reduce the number of reported VUS for TBD patients.

## Introduction

Telomeres at the end of human chromosomes protect the genome from degradation by interacting with the shelterin complex. The telomeres gradually shorten during cell replication due to the inability of the DNA polymerase to fully copy the chromosome ends. As a result, the protective telomere structure cannot be maintained, and the cell will eventually enter senescence and/or apoptosis^1,2^. A selection of highly proliferating cells, including germ cells, hematopoietic stem cells, and activated lymphocytes, counteract the telomere attrition by expressing the enzyme telomerase. Telomerase is a reverse transcriptase enzyme encoded by *TERT* (telomerase reverse transcriptase) and *TERC* (telomerase RNA component) that associates with several proteins essential for stability and telomere binding, such as DKC1, NHP2, NOP10, GAR1, and TCAB1^3,4^. Variants in telomere-associated genes can lead to pathological telomere shortening, resulting in telomere biology disorders (TBD)^5^. TBDs are usually characterized by short telomere length combined with hematological and/or pulmonary symptoms, including bone marrow failure, idiopathic pulmonary fibrosis, and dyskeratosis congenita^6–8^. TBD patients have a heterogenous clinical presentation, and families often display genetic anticipation, which results in earlier onset and more severe manifestation of the disease across generations^9,10^.

Determining the pathogenicity of a novel genetic variant can be challenging, mainly due to variable phenotypic expression and reduced penetrance^11,12^. Support for pathogenicity is for example the absence of the variant in the general population, in silico algorithms predicting a deleterious effect of the variant, and co-segregation of the variant with disease in the family^9,13,14^. Some variants are considered pathogenic or likely pathogenic by consensus guidelines from the American College of Medical Genetics and Genomics (ACMG), but many are classified as variants of uncertain significance (VUS) in the absence of additional supporting evidence of pathogenicity^15^. Identified variants in telomere-associated genes are evaluated using the ACMG guidelines, but may also be supplemented by telomere length analysis or other functional assays^12,16,17^. In addition, variants in genes encoding components of the telomerase enzyme could be evaluated by in vitro assays such as site-directed mutagenesis. This technique involves incorporation of the variant into a plasmid, transformation into a host cell, and then telomerase activity (TA) measurements^18,19^. However, this approach is time-consuming as it requires primer design and sequencing of the transformed cells to confirm the presence of the mutation. In addition, the cellular effects of the variants may not be fully recapitulated by site-directed mutagenesis assays. As an alternative, functional analysis of TA can be performed directly on patient-derived cells which exhibit phenotypes directly related to the disease, offering an advantage in form of clinical relevance.

In this study, we aimed to develop a functional assay for measuring TA in patient-derived cells based on the Telomeric Repeat Amplification Protocol (TRAP) combined with qPCR. To validate the assay, TA was analysed in activated T-cells from healthy controls and from patients with a pathogenic or likely pathogenic variant in a telomerase-associated gene, as these variants are expected to impair the function of the telomerase enzyme. Having established the assay’s ability to detect reduced TA in known pathogenic cases, we plan to use the method in patients with VUS to support pathogenic evaluation.

## Method

### Sample collection

Peripheral venous blood from 100 healthy blood donors (range: 18–73 years, 51% men) were collected at the University Hospital, Umeå, Sweden, between the years 2021 and 2023. The blood samples were anonymized, and the only available information was the donors’ gender, age, and confirmation that they met the eligibility criteria for blood donation according to the hospital’s standard procedures. For TA analysis, peripheral venous blood was collected in sodium heparin tubes. For telomere length analysis, peripheral venous blood was simultaneously collected in EDTA tubes; however, sufficient material was available from only 90 of the 100 donors and telomere length analysis was therefore limited to this subset. For the control group, we aimed to include a uniform distribution of age and gender for every decade, although perfect match was not obtained for the oldest individuals (18–29 years (10 men and 10 women), 30–39 years (10 men and 10 women), 40 − 49 (10 men and 10 women), 50–59 (10 men and 12 women), and > 60 (11 men and 7 women)). The inclusion criteria for patients with suspected TBD were a pathogenic or likely pathogenic variant in a telomerase-associated gene and sufficient material for TA and RTL analysis. Six TBD patients referred to Clinical Genetics, University Hospital in Umeå for genetic testing by Sanger sequencing and/or telomere length measurements by qPCR were included. The patients carried genetic variants that had been classified as pathogenic (*TERT* p.(Arg865His) (*n* = 1)) or likely pathogenic (*TERT* p.(Leu1017_Leu1019del) (*n* = 1), p.(Asp684Gly) (*n* = 1), and p.(Tyr1002Cys) (*n* = 2), and *TERC* n.64G > A (*n* = 1)) according to the ACMG guidelines^15^.

The study was approved by the Regional ethical review board in Umeå (Dnr 2016/258 − 31) and conducted in accordance with the Declaration of Helsinki. Informed consent was obtained from patients and controls.

### T-cell activation and proliferative capacity

Mononuclear cells (MNC) were isolated from peripheral blood using the standard Lymphoprep™ protocol (Stem Cell Technologies, Cambridge, UK). The cells were counted on a HemoCue^®^ WBC DIFF instrument (HemoCue, Ängelholm, SE) and $\:250\times\:{10}^{3}$ cells/ml were stimulated in RPMI-1640 medium with 2.7% phytohemagglutinin (T-cell specific mitogen), 20% FCS, 200 mM L-glutamine, 5000 IU penicillin, and 5000 µg/ml streptomycin. The cell suspensions were cultured at 37 °C and 5% CO_2_ for three days (72–77 h) to reach maximum telomerase activity of T-cells, which has previously been shown to peak around three days of stimulation with phytohemagglutinin^20,21^. The proliferative capacity of the T-cells was evaluated through S-phase fraction (SPF) analysis by flow cytometry.

An aliquot of each sample was diluted to a concentration of 10^6^ cells/ml. The cell nuclei were isolated by incubation in trypsin solution containing Nonidet P40 detergent and spermine tetrachloride. After vortexing and filtration, the nuclei were stained with propidium iodide solution in the dark for a minimum of 30 min at 4 °C^22,23^. The samples were analyzed using a FACSVia™ flow cytometer (BD Biosciences, Franklin Lakes, NJ, US), and DNA histograms were generated. All datasets were analyzed by the ModFit LT™ software v3.0 (Verity Software House, San Diego, CA, USA) to determine the SPF. The cut-off for acceptable runs was > 10,000 modeled events in the G0/G1, G2/M, and S-phase components of all cycling populations.

### Protein concentration measurements

The activated T-cells were lysed in CHAPS lysis buffer (#S7710, Sigma-Aldrich, Saint Louis, MO, US), and an aliquot from each sample was used for protein concentration measurements with the Pierce™ BCA protein assay kit (#23227, ThermoFisher Scientific, Waltham, MA, US). A standard curve from bovine serum albumin with a working range of 20–2000 µg/ml was included in every run. Optical density was measured at 562 nm on a DeNovix DS-11 instrument (DeNovix, Wilmington, DE). All samples were measured in triplicates with the mean value stated as the total protein concentration. The CHAPS lysates were then treated with dithiothreitol (#Y00147, Invitrogen, Waltham, MA, US) and Recombinant RNasin^®^ Ribonuclease Inhibitor (#N2515, Promega, Madison, WI, US) with a working volume of 1 mM dithiothreitol and 1 U/µl RNasin.

### Telomerase activity measurement

TA was assessed with TRAP, which includes telomerase extension, PCR amplification, and quantification^24^ using the Telomerase Activity Quantification qPCR Assay Kit (#8928, ScienCell Research Laboratories, Carlsbad, CA, US). The assay was conducted according to the manufacturer’s instructions, but modified slightly to allow adding a fixed protein concentration instead of a fixed number of cells to the telomerase extension step. A T-lymphoblastoid cell line (CCRF-CEM) was included as a reference in every run to monitor the efficiency of the assay. The CCRF-CEM cells were lysed in CHAPS lysis buffer (Sigma-Aldrich) to an equivalent of 25,000 cells/µl, corresponding to approximately 1000 ng/µl. 2000 ng CCRF-CEM lysate or 500 ng of sample lysate (control, TBD, CCRF-CEM positive control, heat-inactivated CCRF-CEM negative control, or no template control) was added to the telomerase extension buffer in separate test tubes. The tubes were incubated at 37 °C for 3 h on a Veriti™ 96-Well Fast Thermal Cycler (Applied Biosystems, Waltham, MA, US), and thereafter heated to 85 °C for 10 min to inactivate the telomerase enzyme. A standard curve (concentration 100 − 0.16 ng/µL, dilution 1:5) was generated from the reaction with 2000 ng CCRF-CEM to monitor the qPCR runs. The TA of each sample (Control: *n* = 100 and TBD: *n* = 6) was measured in duplicates on a QuantStudio™ 6 Flex System with the QuantStudio™ Real-Time PCR software v1.3 (ThermoFisher Scientific, Waltham, MA, US). The software set an automatic cycle threshold (CT) and baseline for each run. TA was calculated as $\:{2}^{{-(CT}_{X}-{CT}_{P})}$, where CT_X_ corresponds to the mean CT-value of the analyzed sample and CT_P_ to the mean CT-value of the CCRF-CEM positive control. The TRAP assay was run on two separate occasions for sixteen samples (Control: *n* = 10 and TBD: *n* = 6) to monitor assay reproducibility.

### Telomere length measurements

Genomic DNA was extracted from peripheral blood leukocytes from 90 controls and six TBD patients using the Gentra Puregene blood kit (#158023, Qiagen, Hilden, DE) according to the manufacturer’s instructions. Relative telomere length (RTL) was measured by qPCR as previously described^25–27^. Briefly, a telomere to single-copy gene (T/S) ratio was generated for each sample by the formula $\:{\:2}^{{-(CT}_{T}-{CT}_{S})}$, where CT_T_ corresponds to the mean CT-value of the telomere sequence and CT_S_ to the mean value of the single-copy gene. The RTL values were generated by dividing the T/S value of a given sample with the T/S value from a positive control included in every run (CCRF-CEM). A reference curve from CCRF-CEM was included on every PCR plate to monitor the efficiency of the qPCR. All samples were measured in triplicates on two separate occasions, generating a mean RTL from both runs.

#### Statistical analysis

All statistical analyses were conducted in the R statistical software v. 4.2.2^28^. The Mann-Whitney U test was used to compare the controls and TBDs, and linear regression modeling was used to compare continuous variables. To account for SPF-related variation in TA, TA values were first converted back to delta CT (ΔCT) values, where ΔCT = CT_X_-CT_P_. A linear regression model was then fitted to the control cohort, with ΔCT as the dependent variable and SPF as the independent variable. The residuals from this model, representing SPF-adjusted ΔCT values, were centered by adding the predicted ΔCT at the median SPF of the control cohort. Lastly, the adjusted ΔCT values were transformed back to TA values using the $\:{2}^{{-(CT}_{X}-{CT}_{P})}$ formula.

## Results

### TBD patients had short telomeres and reduced telomerase activity but normal proliferative capacity

**Fig. 1:**
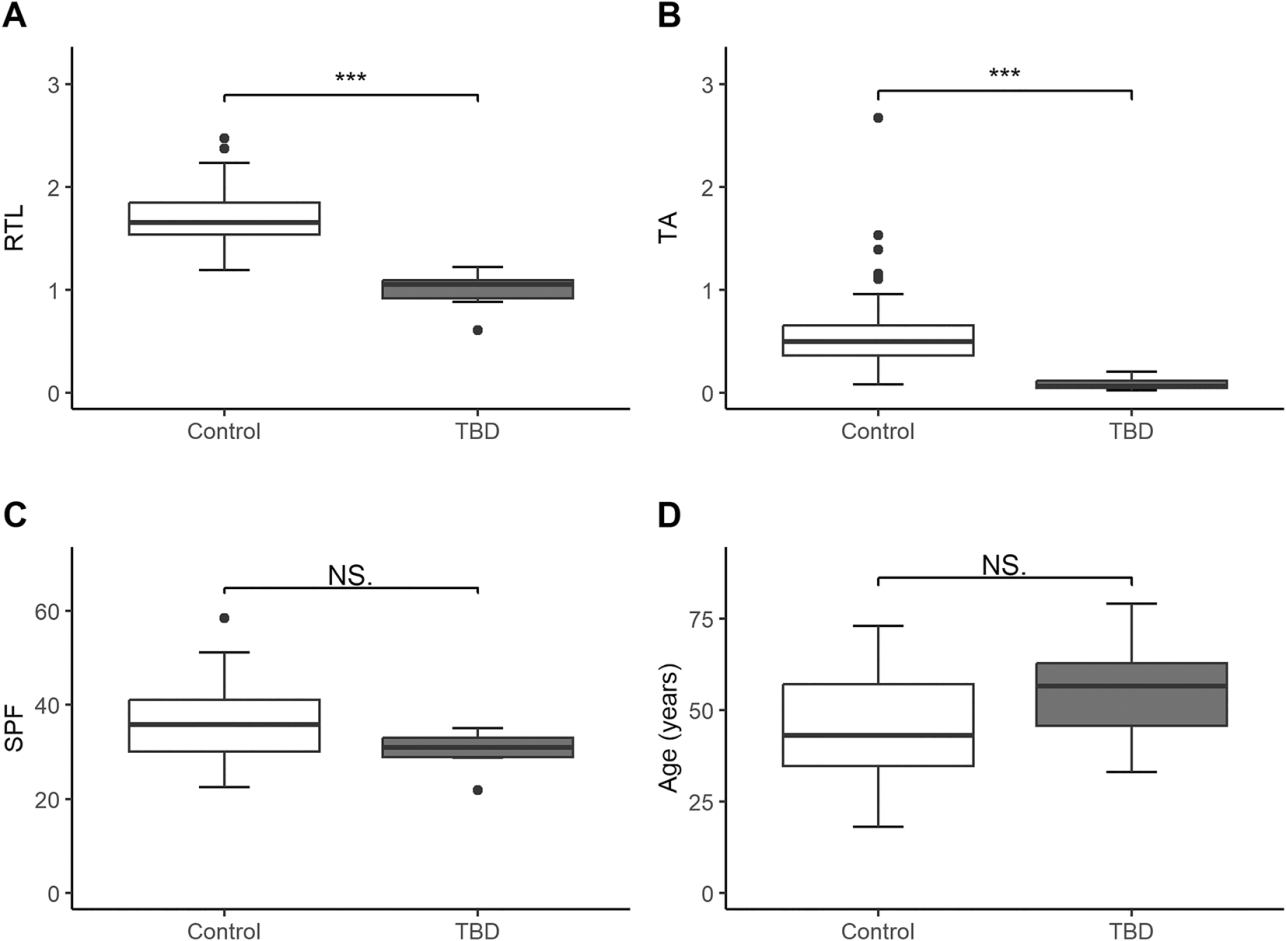
Comparison of relative telomere length (RTL), telomerase activity (TA), S-phase fraction (SPF), and age between controls and TBD patients. ***Corresponds to p-values < 0.001 and NS to not significant (Mann-Whitney U test). (**A**) RTL of leukocytes (Control: *n* = 90 and TBD: *n* = 6). (**B**) TA of T-cells (Control: *n* = 100 and TBD: *n* = 6). (**C**) SPF of T-cells (Control: *n* = 100 and TBD: *n* = 6). (**D**) Age (Control: *n* = 100 and TBD: *n* = 6).

TA was measured in activated T-cells in an adult control cohort (*n* = 100) and evaluated in six TBD patients with a pathogenic or likely pathogenic variant in a telomere-associated gene (*TERT*, *n* = 5 and *TERC*, *n* = 1) (Table 1). The control cohort had a uniform distribution of age and gender across each decade (range: 18–73 years, 51% men), enabling the observation of age-related variation in TA. The activation-induced TA is known to peak at day three of stimulation^20,29^ and our cohort therefore had a median activation time of 72.5 h (range: 72–77 h). Additional blood samples for RTL analysis were available for 90 of the controls and all six TBD patients. We examined whether RTL, TA, SPF, and age differed between the controls and TBDs. There was a significant difference in RTL (Control: 1.69 ± 0.25, TBD: 0.99 ± 0.21, *p* < 0.001) and TA (Control: 0.55 ± 0.34, TBD: 0.09 ± 0.07, *p* < 0.001), between the groups, but no significant difference in SPF (Control: 35.7 ± 7.4, TBD: 30.1 ± 4.7, *p* = 0.060) or age (Control: 44.5 ± 14.3, TBD: 55.3 ± 16.1, *p* = 0.147) (Fig. 1).

**Table 1:** Genetic variants in TBD patients.

TBD sample	Age	Gender	Variant	Symptoms	ACMG classification before TA analysis	RTL	SPF	TA_adj_	SD	Classification after TA analysis
**Sample 1**	59	F	*TERT* p.(Leu1017_Leu1019del)	Pulmonary fibrosis, pancytopenia, early graying of hair, osteoporosis	Likely pathogenic	0.88	29.1	0.27	1–3	Likely pathogenic
**Sample 2**	64	M	*TERT* p.(Asp684Gly)	Pulmonary fibrosis	Likely pathogenic	1.07	32.9	0.15	1–3	Likely pathogenic
**Sample 3**	33	F	*TERT* p.(Tyr1002Cys)	Pulmonary fibrosis, pancytopenia, early graying of hair, liver cirrhosis	Likely pathogenic	1.10	35.0	0.04	> 3	Pathogenic
**Sample 4**	79	F	*TERT* p.(Tyr1002Cys)	Cancer, fragile nails, skin lesions, osteoporosis	Likely pathogenic	1.03	33.0	0.07	> 3	Pathogenic
**Sample 5**	43	M	*TERT* p.(Arg865His)	Pulmonary fibrosis	Pathogenic	1.22	22.0	0.05	> 3	Pathogenic
**Sample 6**	54	M	*TERC* n.64G > A	Pulmonary fibrosis, pancytopenia, renal failure	Likely pathogenic	0.61	28.8	0.09	> 3	Pathogenic

TBD = Telomere biology disorder. ACMG = American College of Medical Genetics and Genomics. RTL = relative telomere length of leukocytes. SPF = S-phase fraction of T-cells. TA_adj_=telomerase activity (TA) adjusted for SPF in T-cells. SD = standard deviation.

### Positive association between telomerase activity and proliferative capacity in control samples

**Fig. 2:**
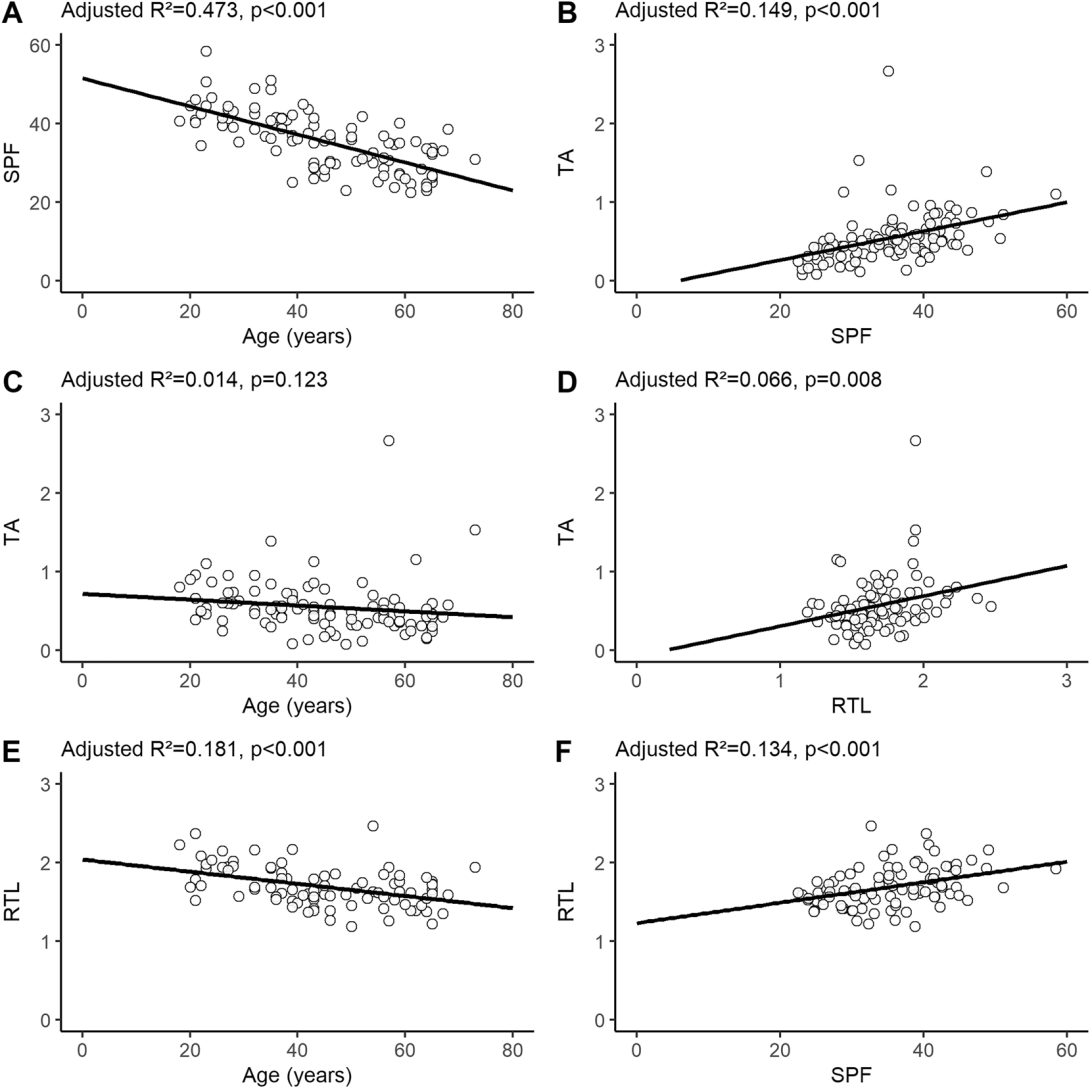
Linear regression analysis of the relationship between S-phase fraction (SPF), telomerase activity (TA), age, and relative telomere length (RTL) in the control cohort. The SPF and TA were measured in activated T-cells (*n* = 100) and RTL in leukocytes (*n* = 90). (**A**) SPF and age. (**B**) TA and SPF. (**C**) TA and age. (**D**) TA and RTL. (**E**) RTL and age. (**F**) RTL and SPF.

Previous studies have reported a decline in TA in normal activated T-cells with age^30^ and a decline in T-cell proliferative capacity with age, which could influence TA^31^. Therefore, we investigated the association between SPF, age, RTL, and TA in the control cohort. The controls showed a moderate negative correlation between SPF and age (adjusted R^2^ = 0.473, *p* < 0.001, Fig. 2A) and a weak positive correlation between SPF and TA (adjusted R^2^ = 0.149, *p* < 0.001, Fig. 2B). However, there was no correlation between TA and age (adjusted R^2^ = 0.014, *p* = 0.123, Fig. 2C). In addition, age-related telomere attrition in T-cells has previously been associated with reduced TA^30^. In our cohort, RTL was measured in leukocytes, as the quantity of available material did not allow for T-cell isolation. Leukocyte RTL had a weak positive correlation with TA (adjusted R^2^ = 0.066, *p* = 0.008, Fig. 2D), a moderate negative correlation with age (adjusted R^2^ = 0.181, *p* < 0.001, Fig. 2E), and a weak positive correlation with SPF (adjusted R^2^ = 0.134, *p* < 0.001, Fig. 2F). In a multiple linear regression analysis, both SPF and age were significantly associated with TA (*p* < 0.001 and *p* = 0.046, respectively), while RTL was not (*p* = 0.068) (Table 2). However, after excluding RTL, age was no longer significantly associated with TA (*p* = 0.075) (Table 2). These results confirmed that the T-cell proliferative capacity declined with age and that a lower proliferative capacity resulted in lower TA.

**Table 2:** Multiple linear regression analysis of the effects of S-phase fraction (SPF), age, and relative telomere length (RTL) on telomerase activity (TA). Significant p-values are indicated by bold text.

Variables	Adjusted *R*^2^	Intercept	Estimate	Standard error	*p*-value
Model 1	0.202	−1.059			
SPF			0.025	0.006	**< 0.001**
AGE			0.007	0.003	**0.046**
RTL			0.271	0.150	0.068
Model 2	0.168	−0.606			
SPF			0.026	0.006	**< 0.001**
Age			0.005	0.003	0.075

### Reduced telomerase activity in TBD

**Fig. 3:**
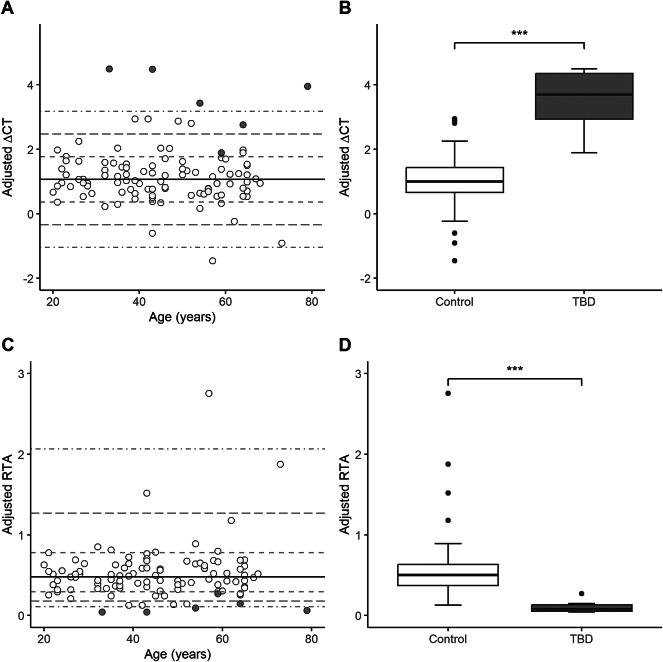
ΔCT and telomerase activity (TA) adjusted for S-phase fraction (SPF) and plotted against age. Controls are represented as white circles and TBD patients as grey circles. ***Corresponds to p-values < 0.001 (Mann-Whitney U test). (**A**) Adjusted ΔCT in controls and TBD plotted against age. The black solid line represents the mean of the normal controls and the black dashed lines represent standard deviation ± 1, ±2, and ± 3. (**B**) Boxplots of adjusted ΔCT in controls and TBD patients. The black solid line represents the median. (**C**) Adjusted TA in controls and TBD plotted against age. The black solid line represents the mean adjusted TA (converted from the mean ΔCT) of the normal controls and the black dashed lines represent standard deviation ± 1, ±2, and ± 3 from the mean (converted from the ΔCT standard deviations). (**D**) Boxplots of adjusted TA in controls and TBD. The black solid line represents the median.

The TA was adjusted for SPF to compensate for age and variation in proliferative response. The TA values were converted to ΔCT values (difference between sample (CT_X_) and the control cell line (CT_P_)), since these values are log-scaled and better reflect the distributional assumptions of linear regression. A linear regression model was constructed using SPF as the independent variable and ΔCT of healthy blood donors as the dependent variable. Positive ΔCT values meant the sample had a higher CT value than the control cell line and thus a lower TA. Plotting the adjusted ΔCT (ΔCT_adj_) values against age showed that the healthy blood donors had a ΔCT_adj_ range of ± 3 standard deviations (SD) (Fig. 3A). Four TBD patients (*TERT* p.(Tyr1002Cys) (*n* = 2), *TERT* p.(Arg865His) (*n* = 1) and *TERC* n.64G > A (*n* = 1)) had ΔCT_adj_ values which were more than 3 SD from the mean of the controls, clearly separating them from the healthy cohort. The other two TBD patients (*TERT* p.(Leu1017_Leu1019del) and *TERT* p.(Asp684Gly)) differed by at least 1 SD. At a group level, the TBD patients had a significantly higher mean ΔCT_adj_ than controls (3.50 versus 1.06, *p* < 0.001, Fig. 3B) and, correspondingly, a lower mean TA_adj_ than the controls (0.11 versus 0.54, *p* < 0.001, Fig. 3C and D) (Table 1).

### Assay reproducibility

**Fig. 4:**
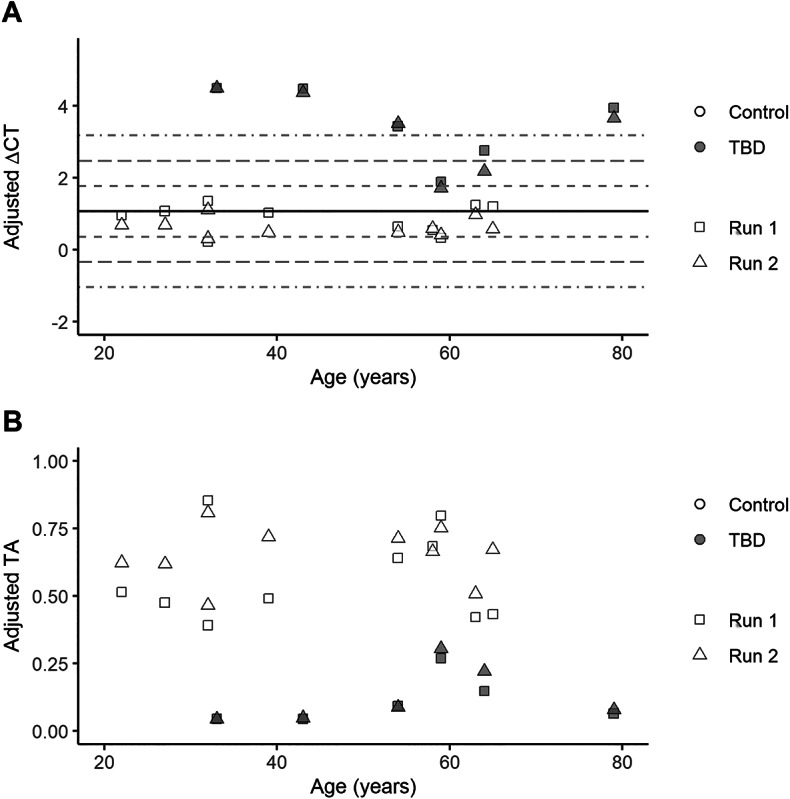
Monitoring telomerase activity (TA) variation between different PCR plates. (**A**) Adjusted ΔCT value for each sample (*n* = 16) on the first and second plate. The black solid line represents the mean adjusted ΔCT and the black dashed lines standard deviation ± 1, ±2, and ± 3 based on the entire control cohort (*n* = 100). (**B**) Adjusted TA for each sample (*n* = 16) on the first and second plate.

To monitor the TA variation with the assay, 16 samples (ten randomly selected controls and all six TBD patients) were run on two separate occasions. The inter-assay coefficient of variation had a median of 10% (4%;15%, 25th;75th percentiles), which was considered acceptable. The ΔCT_adj_ values from both runs were plotted against age (Fig. 4A), showing that there was small variation between the first and second run for most samples. Converting the ΔCT_adj_ to TA_adj_ values showed that lower TA_adj_ values differed less between plates than those of higher TA_adj_ values (Fig. 4B).

In clinical routine, blood samples from patients with suspected TBD may be transported between medical centres. This process can take several days before the samples reach the laboratory, which may have a negative impact on cell viability. In our TBD cohort, MNCs were isolated within 7–32 h of blood withdrawal. To examine if time duration between blood withdrawal and MNC isolation had any preanalytical effect on SPF, three additional blood samples from healthy controls were examined. MNCs were isolated at two time points; immediately and after storage in room temperature for two days. After stimulation, SPF was higher than 30% at both time points for all three samples. No statistical analysis could be conducted due to the low sample size. However, our results indicate that blood samples could be analyzed after two days in room temperature, even though a more thorough study of the preanalytical phase is required.

## Discussion

We have evaluated the relevance of implementing TA measurements as a functional test for pathogenicity assessment of variants in telomere-associated genes. Accurate variant classification relies on many types of evidence to support interpretation^15^. Most disease-associated *TERT* variants observed in TBD patients are missense substitutions and occur within all structural domains^32^. In the absence of compelling family history or supporting functional evidence, most rare variants are classified as VUS and thus present a dilemma, since they would not be actionable in clinical practice. Furthermore, in silico prediction algorithms may have limited utility in predicting the effects of novel missense variants in patients. We therefore determined the functional effect of rare pathogenic and likely pathogenic variants in *TERT* or *TERC*, that were previously identified in TBD patients at our clinic. These variants are expected to impair telomerase function and were included to establish the assay’s ability to detect reduced TA.

It is well-known that telomere length decreases with age in normal blood cells^10,16,33^ but there is conflicting data regarding the association between TA and age in normal activated lymphocytes^30,34,35^. We evaluated if age affected TA in our normal cohort but did not identify a significant association. However, the proliferative capacity of activated T-cells decreased with age, and lower SPF resulted in lower TA. These results are in line with a previous study that found a significant correlation between TA in activated T-cells and proliferative capacity^31^. We therefore suggest that adjustment of proliferative capacity should be done during TA analysis. Although the TBD patients had significantly lower TA than the controls, there was no difference in SPF between the groups, indicating that the reduced TA in TBD patients was not due to reduced proliferative capacity. TA is not required for short-term activation-induced proliferation of normal T-cells^29^ and could explain why there was no significant difference in SPF between controls and TBDs. Furthermore, leukocyte RTL was weakly correlated with TA and was not a significant predictor in our multiple linear regression analysis. Prior research has shown discrepancies in telomere length attrition with age between different cell types, with lymphocytes exhibiting more rapid attrition compared to granulocytes^10^. The weak correlation between TA and leukocyte RTL in our control cohort may be attributed to the different cell populations for each measurement. On the other hand, telomere length in T-cells and other leukocytes is strongly correlated^30,36–38^ and leukocyte RTL thus gives a good representation of T-cell telomere attrition. Ideally, telomere length would have been assessed in T-cells; however, this was not feasible due to limited sample availability. Furthermore, analysis of RNA expression patterns (e.g., *hTERT* and other telomere-associated genes) could have added valuable information, but was not possible due to the limited number of T-cells.

Natural aging causes immunosenescence, which is a gradual decline in immune function where one of the characteristics is a shift in naïve/memory T-cell ratio^33,39^. The shift in T-cell ratio contributes to decreased TA with age, since naïve T-cells respond better to activation than memory cells or effector T-cells^33,40^. In addition, it has been shown that only a fraction of CD28 + T-cells express robust TA upon activation with anti-CD3/CD28^29^. We did not investigate TA in specific T-cell subtypes, and thus, our study cannot confirm if any sub-fraction was more prone to express telomerase. However, the age-related reduction of SPF indicated that our cohort showed the same pattern.

Genetic variants in components of the telomerase enzyme can cause total loss of function, partial loss of function, or have little to no effect^41^. In our analysis, four patients had a TA_adj_ which were more than 3 SD below the mean of controls, which was considered a significant difference and support for pathogenicity. One of these patients carried the pathogenic variant *TERT* p.(Arg865His), which has been demonstrated to have decreased TA compared to wild-type *TERT* in previous in vitro studies^18,19,41^. Two patients carried a novel *TERT* p.(Tyr1002Cys) variant and one patient a novel *TERC* n.64G > A variant that has not been previously described. Since these patients had short telomeres and a TA_adj_ at the same level as the known *TERT* p.(Arg865His), we considered these novel variants to be pathogenic. The remaining two TBD patients had a TA_adj_ which was less than 3 SD below the mean of controls and was considered less significant, thus supporting pathogenicity at a lower level of evidence. These variants should not be reclassified^15,17^. Further evaluation of pathogenic and likely pathogenic variants is warranted to confirm these thresholds, as well as to determine whether the functional evidence should be considered strong, moderate, or weak, according to the ACMG consensus guidelines. However, these variants are essential for establishing a reference for what constitutes pathogenic TA levels and once this baseline is defined, VUS can be assessed. Support for reclassification of VUS to pathogenic would be TA levels comparable of known pathogenic variants. Conversely, TA levels comparable to controls would argue against pathogenicity. Assay reproducibility is a requirement for implementing TA analysis as an evaluation tool. Reanalysis of ten controls and all six TBDs showed small variation and identical classification between the first and second run, indicating that the assay produces consistent and reliable results. However, further studies involving repeated testing of samples over time are required to determine the maximum allowable duration before lymphocyte stimulation must be initiated following blood collection.

In our cohort, genetic variants in only *TERT* and *TERC* were analyzed. These are the main components of the telomerase enzyme, and changes in these genes are expected to alter the enzymatic effect^5,42^. Variants in genes encoding for telomerase-accessory proteins (e.g., *DKC1*,* NHP2*,* NOP10*, or *GAR1*) would most likely have the same effect on TA in stimulated T-cells, but this should be confirmed in future studies. The shelterin components (*TRF1*, *TRF2*, *TINF2*, *RAP1*, *POT1*, and *ACD1*) have been linked to TBD and could compromise the function of telomerase at the telomeres^42^. However, the TRAP assay uses an artificial telomeric sequence, and a dysfunctional shelterin complex would probably not have a functional effect on TA with this assay. Thus, stimulation of T-cells from patients with pathogenic genetic variants in the shelterin complex would most likely have normal TA in the assay, but this also needs further evaluation.

In conclusion, we have shown that activated T-cells from TBD patients with a disease-causing variant had reduced telomerase activity. Pathogenicity was supported for variants with a TA that differed more than 3 SD from normal controls. We suggest that this functional assay of TA can provide a valuable tool to aid in clinical annotation of VUS and thus reduce the number of reported uncertain variants.

## Data Availability

The dataset generated and analyzed during the current study are available from the corresponding author on reasonable request.

## References

[1] de Lange, T. Shelterin-Mediated telomere protection. *Annu. Rev. Genet.* **52**, 223–247. 10.1146/annurev-genet-032918-021921 (2018). 30208292 10.1146/annurev-genet-032918-021921

[2] Rossiello, F., Jurk, D. & Passos, J. F. D’Adda Di fagagna, F. Telomere dysfunction in ageing and age-related Diseases. *Nat. Cell. Biol.* **24**, 135–147. 10.1038/s41556-022-00842-x (2022). 35165420 10.1038/s41556-022-00842-x PMC8985209

[3] Shay, J. W. & Wright, W. E. Telomeres and telomerase: three decades of progress. *Nat. Rev. Genet.* **20**, 299–309. 10.1038/s41576-019-0099-1 (2019). 30760854 10.1038/s41576-019-0099-1

[4] Blackburn, E. H., Epel, E. S. & Lin, J. Human telomere biology: A contributory and interactive factor in aging, disease risks, and protection. *Science* **350**, 1193–1198. 10.1126/science.aab3389 (2015). 26785477 10.1126/science.aab3389

[5] Armando, R. G., Gomez, M., Maggio, D. L., Sanmartin, J., Gomez, D. E. & M. C. & Telomeropathies: etiology, diagnosis, treatment and follow-up. Ethical and legal considerations. *Clin. Genet.* **96**, 3–16. 10.1111/cge.13526 (2019). 30820928 10.1111/cge.13526

[6] Armanios, M. Telomerase and idiopathic pulmonary fibrosis. *Mutat. Res.* **730**, 52–58. 10.1016/j.mrfmmm.2011.10.013 (2012). 22079513 10.1016/j.mrfmmm.2011.10.013 PMC3292861

[7] Calado, R. T. et al. Constitutional hypomorphic telomerase mutations in patients with acute myeloid leukemia. *Proc. Natl. Acad. Sci. U S A*. **106**, 1187–1192. 10.1073/pnas.0807057106 (2009). 19147845 10.1073/pnas.0807057106 PMC2627806

[8] Vulliamy, T., Marrone, A., Dokal, I. & Mason, P. J. Association between aplastic anaemia and mutations in telomerase RNA. *Lancet* **359**, 2168–2170. 10.1016/s0140-6736(02)09087-6 (2002). 12090986 10.1016/S0140-6736(02)09087-6

[9] Vulliamy, T. et al. Disease anticipation is associated with progressive telomere shortening in families with dyskeratosis congenita due to mutations in TERC. *Nat. Genet.* **36**, 447–449. 10.1038/ng1346 (2004). 15098033 10.1038/ng1346

[10] Aubert, G., Baerlocher, G. M., Vulto, I., Poon, S. S. & Lansdorp, P. M. Collapse of telomere homeostasis in hematopoietic cells caused by heterozygous mutations in telomerase genes. *PLoS Genet.* **8**, e1002696. 10.1371/journal.pgen.1002696 (2012). 22661914 10.1371/journal.pgen.1002696 PMC3355073

[11] Xin, Z. T. et al. Functional characterization of natural telomerase mutations found in patients with hematologic disorders. *Blood* **109**, 524–532. 10.1182/blood-2006-07-035089 (2007). 16990594 10.1182/blood-2006-07-035089

[12] Norberg, A. et al. Novel variants in nordic patients referred for genetic testing of telomere-related disorders. *Eur. J. Hum. Genet.* **26**, 858–867. 10.1038/s41431-018-0112-8 (2018). 29483670 10.1038/s41431-018-0112-8 PMC5974393

[13] Du, H. Y. et al. TERC and TERT gene mutations in patients with bone marrow failure and the significance of telomere length measurements. *Blood* **113**, 309–316. 10.1182/blood-2008-07-166421 (2009). 18931339 10.1182/blood-2008-07-166421 PMC2615648

[14] Savage, S. A. Beginning at the ends: telomeres and human disease. *F1000Res* 7 (2018). 10.12688/f1000research.14068.1 10.12688/f1000research.14068.1 PMC5931273 29770205

[15] Richards, S. et al. Standards and guidelines for the interpretation of sequence variants: a joint consensus recommendation of the American college of medical genetics and genomics and the association for molecular pathology. *Genet. Med.* **17**, 405–424. 10.1038/gim.2015.30 (2015). 25741868 10.1038/gim.2015.30 PMC4544753

[16] Alder, J. K. et al. Diagnostic utility of telomere length testing in a hospital-based setting. *Proc. Natl. Acad. Sci. U S A*. **115**, E2358–e2365. 10.1073/pnas.1720427115 (2018). 29463756 10.1073/pnas.1720427115 PMC5877993

[17] Nelson, N. et al. Functional genomics for curation of variants in telomere biology disorder associated genes: A systematic review. *Genet. Med.* **25**, 100354. 10.1016/j.gim.2022.11.021 (2023). 36496180 10.1016/j.gim.2022.11.021

[18] Tsang, A. R., Wyatt, H. D., Ting, N. S. & Beattie, T. L. hTERT mutations associated with idiopathic pulmonary fibrosis affect telomerase activity, telomere length, and cell growth by distinct mechanisms. *Aging Cell.* **11**, 482–490. 10.1111/j.1474-9726.2012.00810.x (2012). 22364217 10.1111/j.1474-9726.2012.00810.x

[19] Tsakiri, K. D. et al. Adult-onset pulmonary fibrosis caused by mutations in telomerase. *Proc. Natl. Acad. Sci. U S A*. **104**, 7552–7557. 10.1073/pnas.0701009104 (2007). 17460043 10.1073/pnas.0701009104 PMC1855917

[20] de Punder, K., Heim, C., Przesdzing, I., Wadhwa, P. D. & Entringer, S. Characterization in humans of in vitro leucocyte maximal telomerase activity capacity and association with stress. *Philos. Trans. R Soc. Lond. B Biol. Sci.* **373**10.1098/rstb.2016.0441 (2018). 10.1098/rstb.2016.0441 PMC5784061 29335365

[21] Yamada, O., Motoji, T. & Mizoguchi, H. Up-regulation of telomerase activity in human lymphocytes. *Biochim. Biophys. Acta*. **1314**, 260–266. 10.1016/s0167-4889(96)00104-8 (1996). 8982280 10.1016/s0167-4889(96)00104-8

[22] Vindeløv, L. L., Christensen, I. J. & Nissen, N. I. A detergent-trypsin method for the Preparation of nuclei for flow cytometric DNA analysis. *Cytometry* **3**, 323–327. 10.1002/cyto.990030503 (1983). 6188586 10.1002/cyto.990030503

[23] Patthey, A. et al. Combination of aneuploidy and high S-phase fraction indicates increased risk of relapse in stage I endometrioid endometrial carcinoma. *Acta Oncol.* **60**, 1218–1224. 10.1080/0284186x.2021.1939146 (2021). 34156893 10.1080/0284186X.2021.1939146

[24] Kim, N. W. et al. Specific association of human telomerase activity with immortal cells and cancer. *Science* **266**, 2011–2015. 10.1126/science.7605428 (1994). 7605428 10.1126/science.7605428

[25] Cawthon, R. M. Telomere measurement by quantitative PCR. *Nucleic Acids Res.* **30**, e47. 10.1093/nar/30.10.e47 (2002). 12000852 10.1093/nar/30.10.e47 PMC115301

[26] Carlund, O. et al. DNA methylation variations and epigenetic aging in telomere biology disorders. *Sci. Rep.* **13**, 7955. 10.1038/s41598-023-34922-1 (2023). 37193737 10.1038/s41598-023-34922-1 PMC10188573

[27] Carlund, O. et al. Semimethylation is a feature of diffuse large B-cell lymphoma, and subgroups with poor prognosis are characterized by global hypomethylation and short telomere length. *Clin. Epigenetics*. **16**, 68. 10.1186/s13148-024-01680-4 (2024). 38773655 10.1186/s13148-024-01680-4 PMC11110316

[28] R: A Language and Environment for Statistical Computing. R Foundation for Statistical Computing, Vienna, Austria, (2022).

[29] Huang, E. E. et al. The maintenance of telomere length in CD28 + T cells during T lymphocyte stimulation. *Sci. Rep.* **7**, 6785. 10.1038/s41598-017-05174-7 (2017). 28754961 10.1038/s41598-017-05174-7 PMC5533788

[30] Lin, Y. et al. Age-associated telomere attrition of lymphocytes in vivo is co-ordinated with changes in telomerase activity, composition of lymphocyte subsets and health conditions. *Clin. Sci. (Lond)*. **128**, 367–377. 10.1042/cs20140481 (2015). 25317735 10.1042/CS20140481 PMC5421624

[31] Tedone, E. et al. Telomere length and telomerase activity in T cells are biomarkers of high-performing centenarians. *Aging Cell.* **18**, e12859. 10.1111/acel.12859 (2019). 30488553 10.1111/acel.12859 PMC6351827

[32] Podlevsky, J. D., Bley, C. J., Omana, R. V., Qi, X. & Chen, J. J. The telomerase database. *Nucleic Acids Res.* **36**, D339–343. 10.1093/nar/gkm700 (2008). 18073191 10.1093/nar/gkm700 PMC2238860

[33] Chebly, A., Khalil, C., Kuzyk, A., Beylot-Barry, M. & Chevret, E. T-cell lymphocytes’ aging clock: telomeres, telomerase and aging. *Biogerontology* **25**, 279–288. 10.1007/s10522-023-10075-6 (2024). 37917220 10.1007/s10522-023-10075-6

[34] Son, N. H., Murray, S., Yanovski, J., Hodes, R. J. & Weng, N. Lineage-specific telomere shortening and unaltered capacity for telomerase expression in human T and B lymphocytes with age. *J. Immunol.* **165**, 1191–1196. 10.4049/jimmunol.165.3.1191 (2000). 10903716 10.4049/jimmunol.165.3.1191

[35] Hiyama, K. et al. Activation of telomerase in human lymphocytes and hematopoietic progenitor cells. *J. Immunol.* **155**, 3711–3715 (1995). 7561072

[36] Lin, J. et al. Systematic and cell Type-Specific telomere length changes in subsets of lymphocytes. *J. Immunol. Res.* **2016** (5371050). 10.1155/2016/5371050 (2016). 10.1155/2016/5371050 PMC4764743 26977417

[37] Lin, J. et al. Analyses and comparisons of telomerase activity and telomere length in human T and B cells: insights for epidemiology of telomere maintenance. *J. Immunol. Methods*. **352**, 71–80. 10.1016/j.jim.2009.09.012 (2010). 19837074 10.1016/j.jim.2009.09.012 PMC3280689

[38] Kimura, M. et al. Synchrony of telomere length among hematopoietic cells. *Exp. Hematol.* **38**, 854–859. 10.1016/j.exphem.2010.06.010 (2010). 20600576 10.1016/j.exphem.2010.06.010 PMC3142672

[39] Liu, Z. et al. Immunosenescence: molecular mechanisms and diseases. *Signal. Transduct. Target. Ther.* **8**, 200. 10.1038/s41392-023-01451-2 (2023). 37179335 10.1038/s41392-023-01451-2 PMC10182360

[40] Patrick, M. & Weng, N. P. Expression and regulation of telomerase in human T cell differentiation, activation, aging and diseases. *Cell. Immunol.* **345**, 103989. 10.1016/j.cellimm.2019.103989 (2019). 31558266 10.1016/j.cellimm.2019.103989 PMC6873926

[41] Zaug, A. J., Crary, S. M., Jesse Fioravanti, M., Campbell, K. & Cech, T. R. Many disease-associated variants of hTERT retain high telomerase enzymatic activity. *Nucleic Acids Res.* **41**, 8969–8978. 10.1093/nar/gkt653 (2013). 23901009 10.1093/nar/gkt653 PMC3799428

[42] Kam, M. L. W., Nguyen, T. T. T. & Ngeow, J. Y. Y. Telomere biology disorders. *NPJ Genom Med.* **6**, 36. 10.1038/s41525-021-00198-5 (2021). 34050178 10.1038/s41525-021-00198-5 PMC8163767

